# Supplementary material for: Modified CLEC3A-Derived Antimicrobial Peptides Lead to Enhanced Antimicrobial Activity against Drug-Resistant Bacteria
Source: Antibiotics (Basel). 2023 Oct 11;12(10):1532. doi: 10.3390/antibiotics12101532 (PMC10604565; doi:10.3390/antibiotics12101532)
Supplement: Supplementary file 1 [file antibiotics-12-01532-s001.zip › antibiotics-2632762-supplementary.pdf]

Modified CLEC3A-derived Antimicrobial Peptides Lead to Enhanced Antimicrobial Activity against Drug-resistant Bacteria

Denise Meinberger[1], Marco G. Drexelius[2,3], Joshua Grabeck[2,3], Gabriele Hermes[1], Annika Roth[1], Dzemal Elezagic[1], Ines Neundorf[2,3], Thomas Streichert[1], Andreas R. Klatt[1,*].

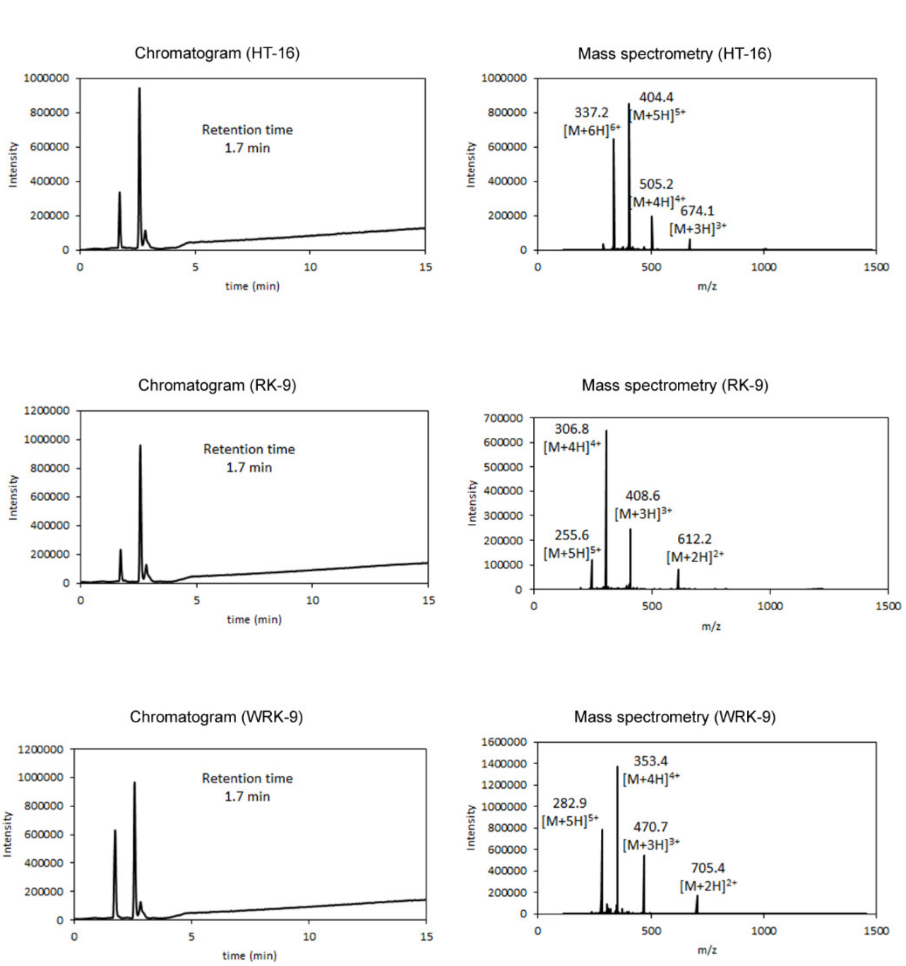

Supplementary Figure S1 Chromatogram (left) and mass spectrometry data (right) verifying the identity of the peptides after synthesis. Diagrams for HT-16 (**A**) and its derived modified peptides RK-9 (**B**), and WRK-9 (**C**)

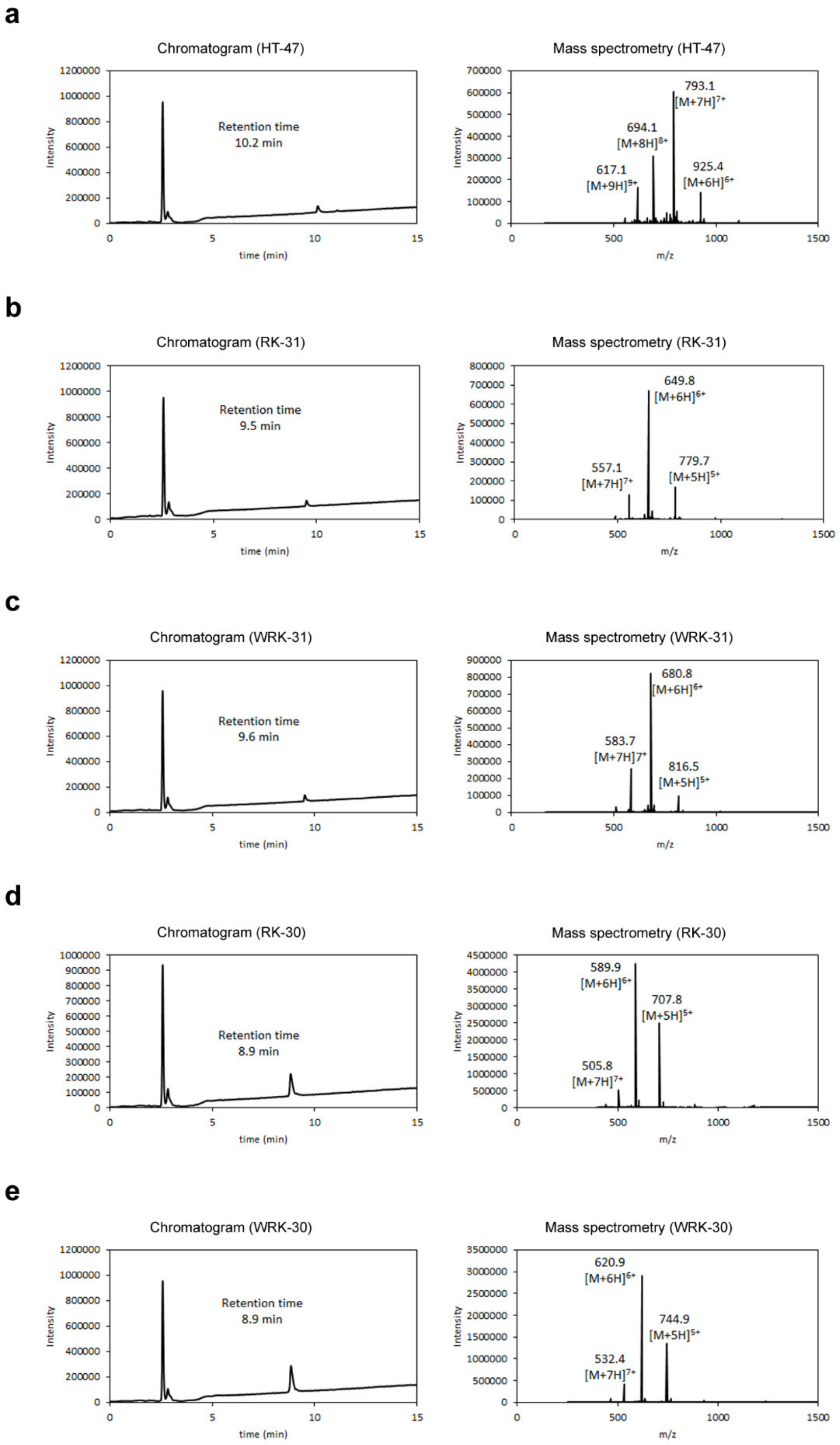

Supplementary Figure S2 Chromatogram (left) and mass spectrometry data (right) verifying the identity of the peptides after synthesis. Diagrams for HT-47 (**A**) and its derived modified peptides RK-31 (**B**), WRK-31 (**C**), RK-30 (**D**), and WRK-30 (**E**)

Supplementary Table S1 Peptide concentrations used in the antimicrobial activity assay

Bacterial strain	Peptides	Used peptide concentrations (µM)									
Ps. aeruginosa	LL-37, ST-16 (Ctrl 1), HT-16, DK-29 (Ctrl 2), HT-47, RK-9, WRK-9, RK-31, WRK-31, RK-30, WRK-30, dWRK-30	5	2.5	1.25	0.63	0.31	0.16	0.08	0.04	0.02	0.01
S. aureus	LL-37, ST-16 (Ctrl 1), HT-16, DK-29 (Ctrl 2), HT-47, RK-9, WRK-9, RK-31, WRK-31, RK-30, WRK-30, dWRK-30	5	2.5	1.25	0.63	0.31	0.16	0.08	0.04	0.02	0.01
MRSA	LL-37, ST-16 (Ctrl 1), HT-16, DK-29 (Ctrl 2), HT-47, RK-9, WRK-9, RK-31, WRK-31, RK-30, WRK-30, dWRK-30	10	5	2.5	1.25	0.63	0.31	0.16	0.08	0.04	0.02
	LL-37, DK-29 (Ctrl 2), WRK-30, dWRK-30	5	2.5	1.25	0.63	0.31	0.16	0.08	0.04	0.02	0.01

Supplementary Table S2 Peptide concentrations used in the cytotoxicity assay

Incubation time (h)	Peptides	Used peptide concentrations (µM)		
24	LL-37, ST-16 (Ctrl 1), HT-16, DK-29 (Ctrl 2), HT-47, RK-9, WRK-9, RK-30, WRK-30	0.3	3	30
	LL-37, DK-29 (Ctrl 2), HT-47, WRK-30, dWRK-30	40	60	80
96	LL-37, ST-16 (Ctrl 1), HT-16, DK-29 (Ctrl 2), HT-47, RK-9, WRK-9, RK-30, WRK-30	0.3	3	30

Supplementary Table S3 Time points used for taking samples in the Biostability assay

Peptide	Time points for sampling			
**HT-47**	5 min	10 min	6 h	8 h
**WRK-30**	5 min	10 min	30 min	1 h
**dWRK-30**	5 min	10 min	24 h	96 h

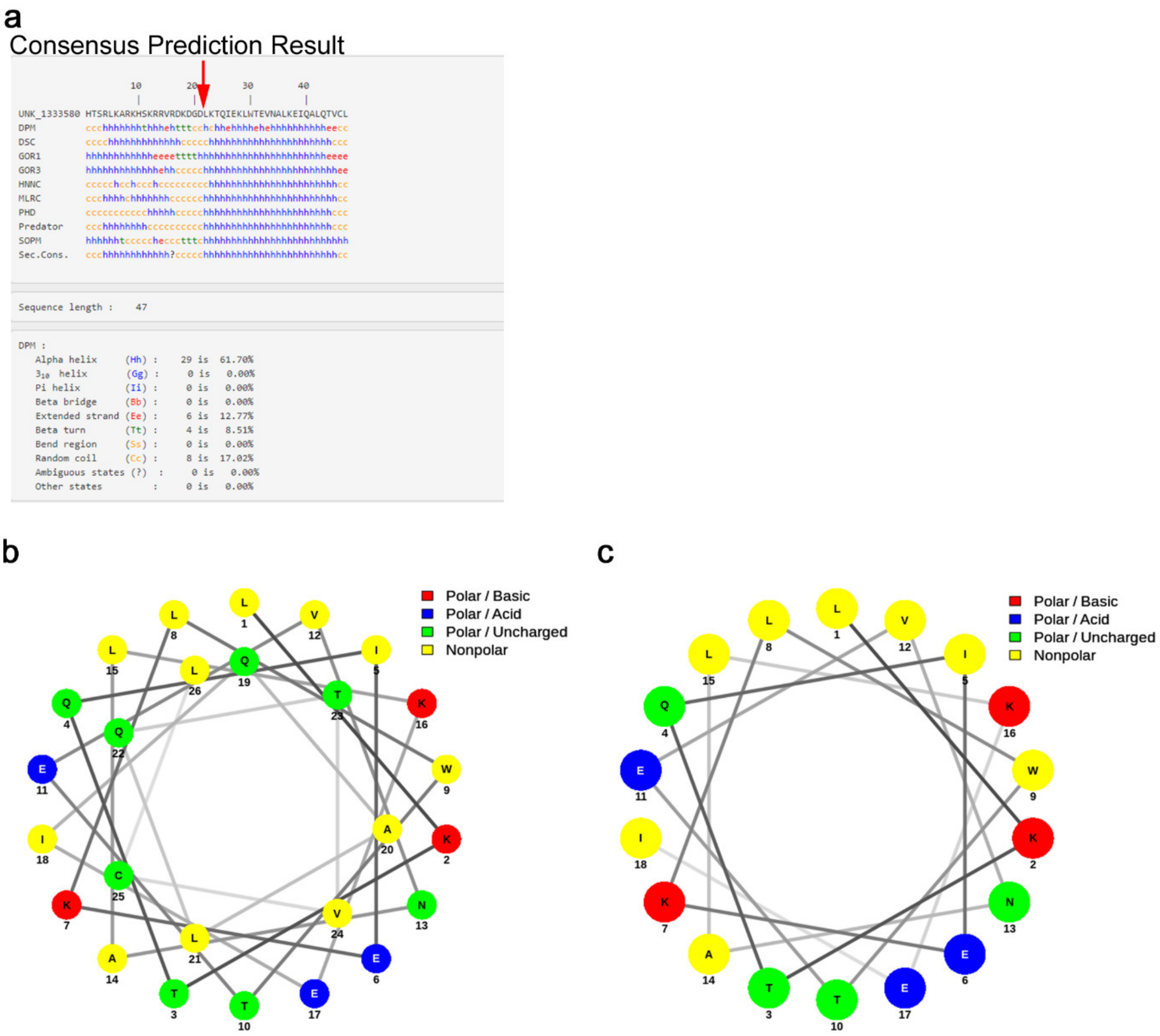

Supplementary Figure S3 Structural prediction of the α-helical organization. (**A**) Consensus Prediction Result of HT-47 generated with NPS@: Network Protein Sequence Analysis [21] using various algorithms. The red arrow indicates the most commonly predicted position for the beginning of the C-Terminal α-helix. (**B**) Helical-wheel-projection of the C-Terminal α-helix of HT-47 starting with L22. (**C**) Helical-wheel-projection of the C-Terminal α-helix of RK-31, WRK-31, RK-30, and WRK-30 derived from the C-Terminal α-helix of HT-47 starting with L22. (http://lbqp.unb.br/NetWheels)

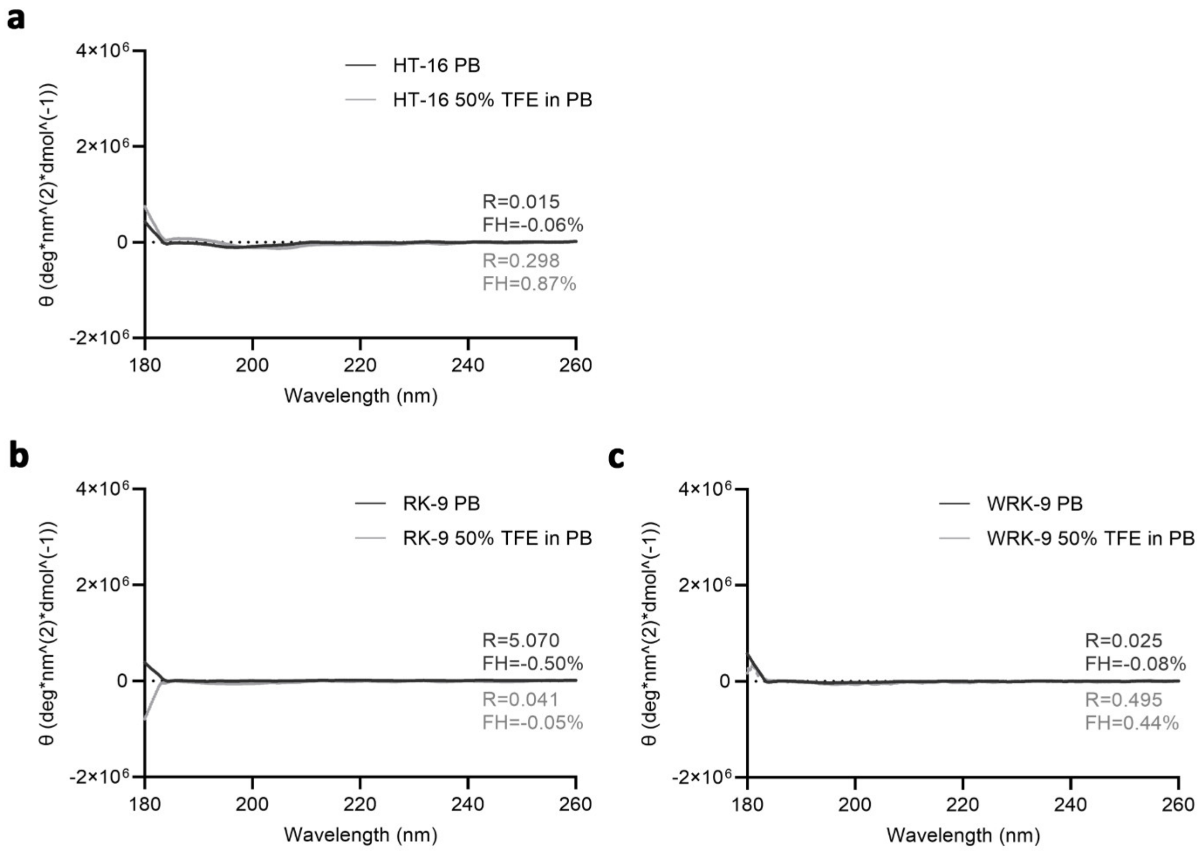

Supplementary Figure S4 Circular dichroism (CD) spectroscopy of CLEC3A-derived peptides HT-16, RK-9, and WRK-9. (**a**) CD-spectroscopy of HT-16 dissolved in phosphate buffer (PB) (black) and 50% TFE in PB (dark grey). (**b**) CD-spectroscopy of RK-9 dissolved in phosphate buffer (PB) (black) and 50% TFE in PB (dark grey). (**c**) CD-spectroscopy of WRK-9 dissolved in phosphate buffer (PB) (black) and 50% TFE in PB (dark grey). R-values were calculated by forming the ratio of the molar ellipticity values at 222 nm and 207 nm. R-values of R=1 describe a perfectly formed α-helix. The fractional helicity (FH) of the peptides was calculated from the molar ellipticity values at 222 nm as described in the methods section. All CD-spectroscopy experiments were performed three times (n=3).

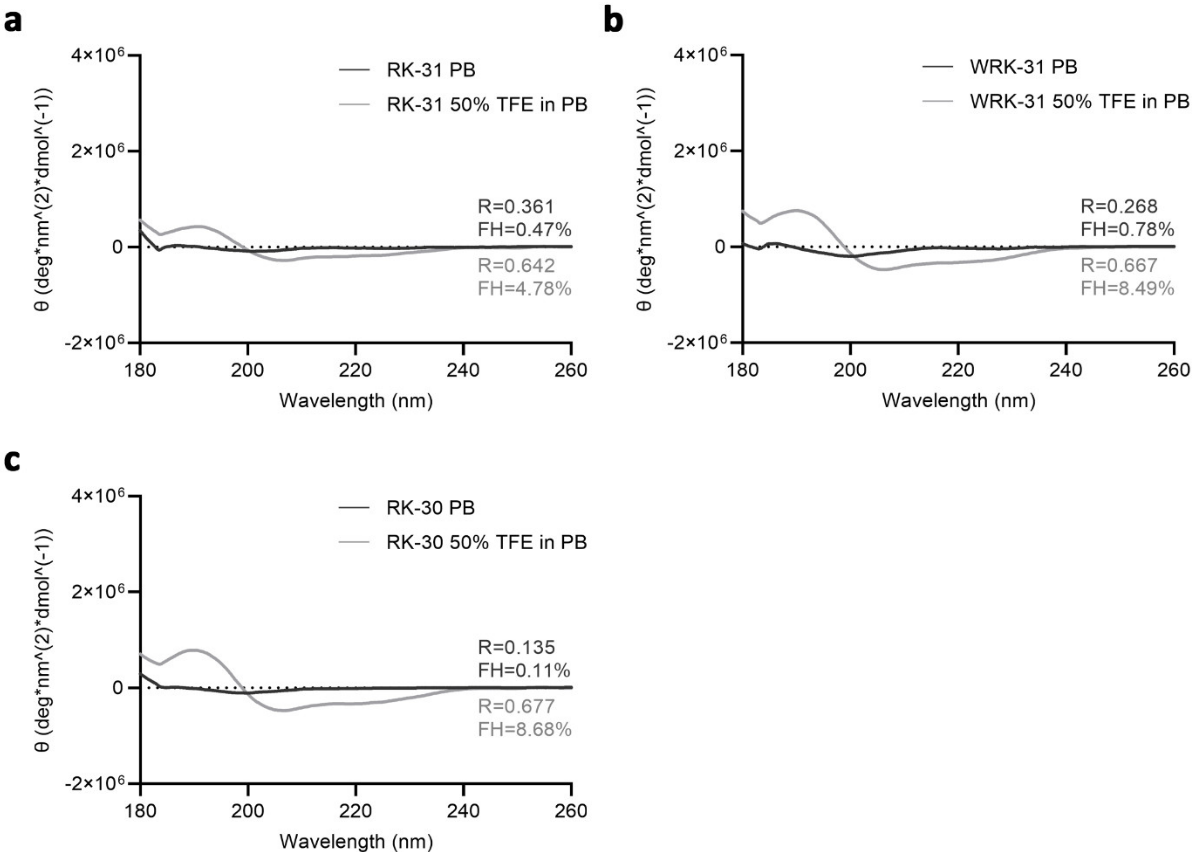

Supplementary Figure S5 Circular dichroism (CD) spectroscopy of CLEC3A-derived peptides RK-31, WRK-31, and RK-30. (**a**) CD-spectroscopy of RK-31 dissolved in phosphate buffer (PB) (black) and 50% TFE in PB (dark grey). (**b**) CD-spectroscopy of WRK-31 dissolved in phosphate buffer (PB) (black) and 50% TFE in PB (dark grey). (**c**) CD-spectroscopy of RK-30 dissolved in phosphate buffer (PB) (black) and 50% TFE in PB (dark grey). R-values were calculated by forming the ratio of the molar ellipticity values at 222 nm and 207 nm. R-values of R=1 describe a perfectly formed α-helix. The fractional helicity (FH) of the peptides was calculated from the molar ellipticity values at 222 nm as described in the methods section. All CD-spectroscopy experiments were performed three times (n=3).

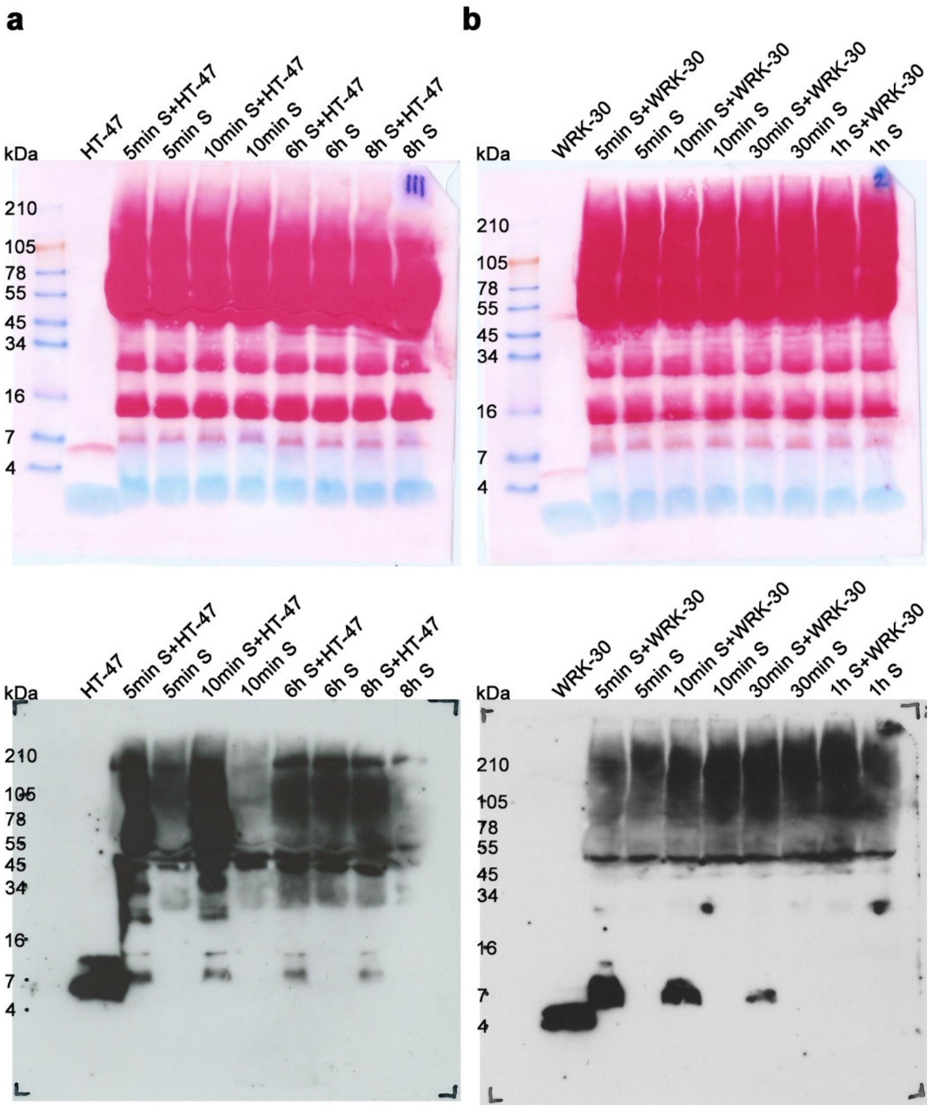

Supplementary Figure S6 Ponceau and immunoblots of the biostability assays of HT-47 (A) and WRK-30 (B). (**A**) Immunoblot of HT-47 samples incubated in murine serum (S) for 5 min, 10 min, 6 h, and 8 h using an α–WRK-30 antibody for detection. A sample of only HT-47 in water and samples of serum (S) without the peptide incubated for the same time periods were used as controls. The figure shows an exposure time of 1 min with representative ponceau (top) and antibody (bottom) stainings in their uncropped, full size. (**B**) Immunoblot of WRK-30 samples incubated in murine serum (S) for 5 min, 10 min, 30 min, and 1 h using an α –WRK-30 antibody for detection. A sample of only WRK-30 in water and samples of serum (S) without the peptide incubated for the same time periods were used as controls. The figure shows an exposure time of 10 min (representative (n=3) ponceau (top) and antibody (bottom) stainings in their uncropped, full size)

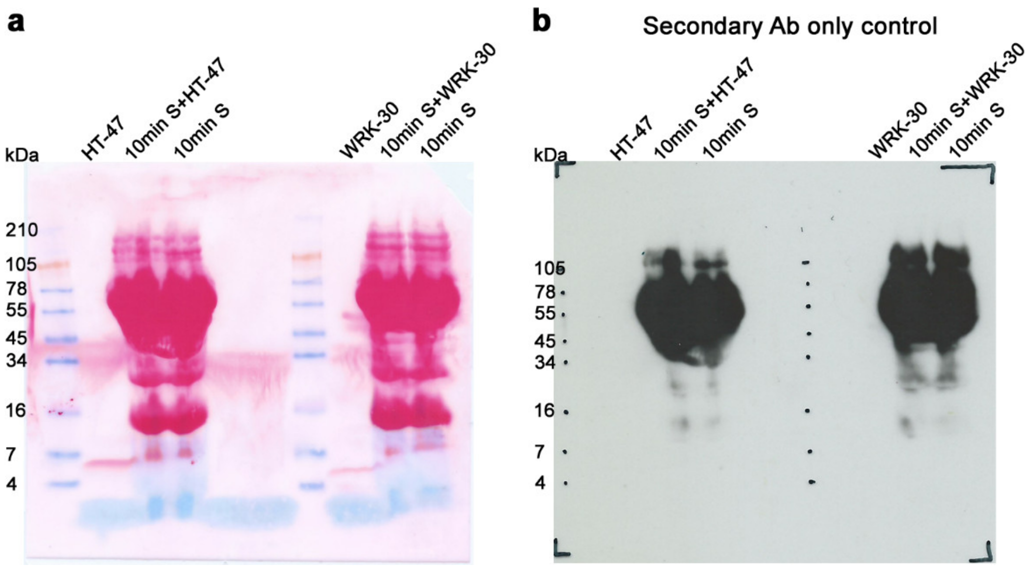

Supplementary Figure S7 Ponceau and immunoblots of the secondary antibody (Ab) only control of the biostability assays. Immunoblot of HT-47 and WRK-30 samples incubated in murine serum (S) for 10 min using an α–rabbit IgG antibody for detection. A sample of only HT-47 and WRK-30 in water and samples of serum (S) without the peptide incubated for the same time periods were used as controls. The figure shows an exposure time of 10 min: (**A**) ponceau and (**B**) antibody stainings of an n=1 in their uncropped, full size

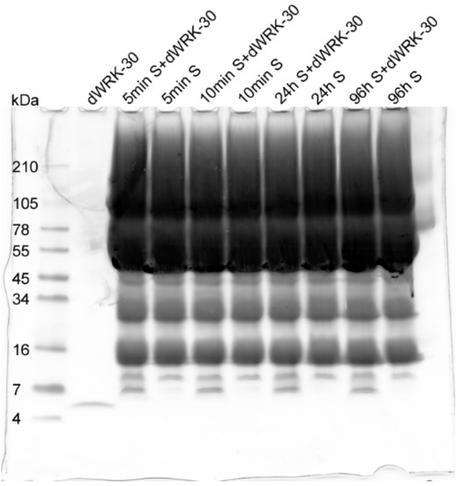

Supplementary Figure S8 Coomassie staining of the biostability assays for dWRK-30. SDS-PAGE of dWRK-30 samples incubated in murine serum (S) for 5 min, 10 min, 24 h, and 96 h using Coomassie brilliant blue for detection. A sample of only dWRK-30 in water and samples of serum (S) without the peptide incubated for the same time periods were used as controls. (**A**). The figure shows a representative SDS-PAGE stained with Coomassie in its uncropped, full size
